# Effect of Polymer Hydrophobicity in the Performance of Hybrid Gel Gas Sensors for E-Noses

**DOI:** 10.3390/s23073531

**Published:** 2023-03-28

**Authors:** Ana Rita Oliveira, Henrique M. A. Costa, Efthymia Ramou, Susana I. C. J. Palma, Ana Cecília A. Roque

**Affiliations:** 1 Associate Laboratory i4HB—Institute for Health and Bioeconomy, School of Science and Technology, NOVA University Lisbon, 2829-516 Caparica, Portugal; ar.oliveira@outlook.pt (A.R.O.); hma.costa@campus.fct.unl.pt (H.M.A.C.); e.ramou2@hull.ac.uk (E.R.); s.palma@fct.unl.pt (S.I.C.J.P.); 2 UCIBIO—Applied Molecular Biosciences Unit, Department of Chemistry, School of Science and Technology, NOVA University Lisbon, 2829-516 Caparica, Portugal

**Keywords:** electronic nose, gas sensing, ionogels, humidity, PDMS, gelatin, ionic liquids, liquid crystals

## Abstract

Relative humidity (RH) is a common interferent in chemical gas sensors, influencing their baselines and sensitivity, which can limit the performance of e-nose systems. Tuning the composition of the sensing materials is a possible strategy to control the impact of RH in gas sensors. Hybrid gel materials used as gas sensors contain self-assembled droplets of ionic liquid and liquid crystal molecules encapsulated in a polymeric matrix. In this work, we assessed the effect of the matrix hydrophobic properties in the performance of hybrid gel materials for VOC sensing in humid conditions (50% RH). We used two different polymers, the hydrophobic PDMS and the hydrophilic bovine gelatin, as polymeric matrices in hybrid gel materials containing imidazolium-based ionic liquids, [BMIM][Cl] and [BMIM][DCA], and the thermotropic liquid crystal 5CB. Better accuracy of VOC prediction is obtained for the hybrid gels composed of a PDMS matrix combined with the [BMIM][Cl] ionic liquid, and the use of this hydrophobic matrix reduces the effect of humidity on the sensing performance when compared to the gelatin counterpart. VOCs interact with all the moieties of the hybrid gel multicomponent system; thus, VOC correct classification depends not only on the polymeric matrix used, but also on the IL selected, which seems to be key to achieve VOCs discrimination at 50% RH. Thus, hybrid gels’ tunable formulation offers the potential for designing complementary sensors for e-nose systems operable under different RH conditions.

## 1. Introduction

Electronic-noses (e-noses) are devices that mimic the biological olfactory system, by combining an array of independent semi-selective gas sensors with a signal transduction unit and a pattern-recognition system, allowing the differentiation of distinct odors [1,2,3]. Gas sensors typically alter their physical or chemical properties in the presence of volatile organic compound (VOC) molecules. The most common gas sensors are metal oxide semiconductors (MOS) or conductive polymers, which typically present low VOC-selectivity and are susceptible to fluctuations in surrounding temperature and humidity [4]. Other gas sensing materials can be used including, for example those incorporating biological receptors [5] or liquid crystal molecules [6].

Humidity is a common interferent in gas sensors and it can seriously affect sensors’ performance [7,8,9]. Different approaches are adopted to correct the effect of humidity. One approach is to lower or even remove the humidity content before samples have contact with gas sensors, therefore operating the system in dry conditions. Another alternative is to correct the humidity signal background by signal processing and machine learning approaches. Lastly, a gas sensor’s composition can be altered to decrease humidity interference. This can be achieved by coating sensors with hydrophobic polymers such as polydimethylsiloxane (PDMS) [10], or by tuning the chemical components of the gas sensor itself [11]. In our previous work [11], we reported that the anion moiety of the ionic liquid component of hybrid gels used as gas sensors can tune the sensors’ sensitivity to humidity. In these hybrid gels, a hydrophilic gelatin polymer was used as the matrix to encapsulate liquid crystal–ionic liquid droplets that act as optical probes.

**Figure 1:**
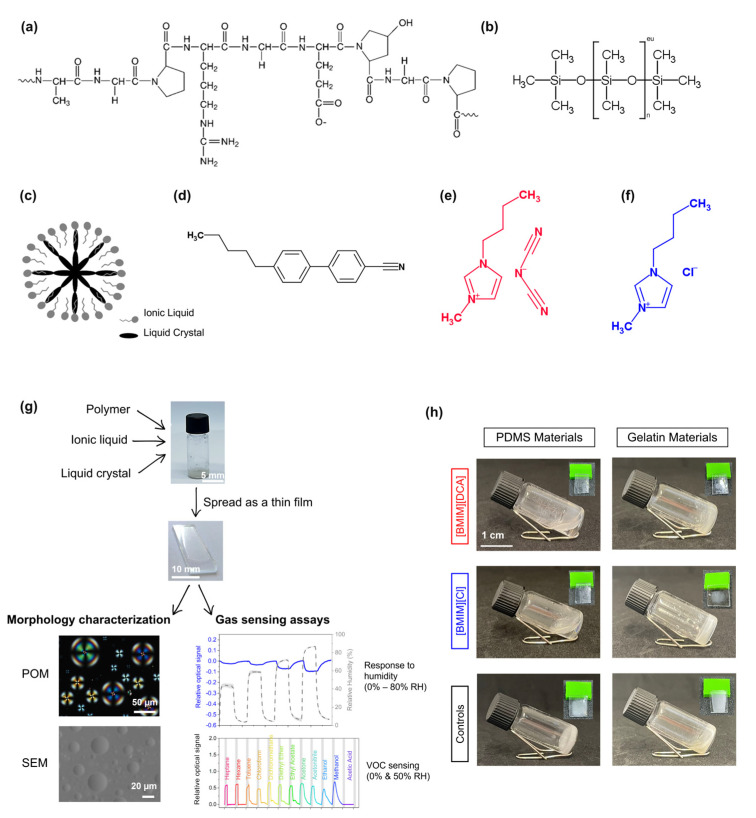
Hybrid gels production and characterization. (**a**) Gelatin and (**b**) PDMS chemical structures. (**c**) Schematic representation of an ionic liquid-liquid crystal-droplet. (**d**) Chemical structure of the liquid crystal 5CB and of (**e**) [BMIM][DCA] and (**f**) [BMIM][Cl] ionic liquids. (**g**) Schematic representation of the method followed for hybrid gels production and characterization. The images are representative of the characterization and sensing assays further detailed in the text. (**h**) Macroscopic appearance of the hybrid gel formulations; the inset photo shows the films produced with each formulation and used as gas sensors.

In the present work, we aim at understanding the effect of the polymer hydrophobicity in hybrid gel gas sensors’ response to humidity. Furthermore, we assess the hybrid gel sensors’ performance to classify different VOCs in a humid atmosphere (average 50% RH). For that, we studied hydrophobic (PDMS) and hydrophilic (bovine gelatin) matrices to incorporate liquid crystal–ionic liquid droplets. We employed the well-studied ionic liquids from the imidazolium family, [BMIM][DCA] and [BMIM][Cl] [11], and the thermotropic liquid crystal 4-cyano-4′-pentylbiphenyl (5CB) which exhibits the nematic phase [12] (Figure 1). The resultant hybrid gels were used as gas sensors in a tailor-made optical e-nose. The sensors’ response to humidity was firstly evaluated, and then their ability to operate under humidified conditions for VOC discrimination. Better VOC classification accuracy in humid conditions was obtained when using the PDMS matrix combined with the [BMIM][Cl] ionic liquid.

## 2. Materials and Methods

### 2.1. Materials

The polymer polydimethylsiloxane (PDMS, Sylgard™ 184 Silicone Elastomer Kit) was purchased from The Dow Chemical Company (Wiesbaden, Germany), and gelatin from bovine skin (gel strength ≈ 225 g; Bloom, Type B) was purchased from Sigma Aldrich (Saint Louis, MO, USA). The liquid crystal 4-cyano-4′-pentylbiphenyl (5CB, >98.0%) was purchased from TCI Europe (Zwijndrecht, Belgium). The ionic liquids 1-butyl-3-methylimidazolium dicyanamide ([BMIM][DCA] > 98.0%) and 1-butyl-3-methylimidazolium chloride ([BMIM][Cl], >98.0%) were purchased from IoLiTec (Heilbronn, Germany). The anhydrous binary salts magnesium chloride (≥98.0%), potassium carbonate (99.9%) and sodium bromide (≥99.0%) were purchased from Sigma-Aldrich (Saint Louis, MO, USA); sodium chloride (>99.5%) and the solvents dichloromethane and hexane were purchased from VWR International (Carnaxide, Portugal). Ethanol and acetic acid (purity 99.8%) were purchased from Sigma-Aldrich (Saint Louis, MO, USA). Acetonitrile (purity 99.9%), chloroform, diethyl ether (HPLC grade), ethyl acetate, heptane, methanol (HPLC grade) and toluene were supplied by Fisher Scientific (Waltham, MA, USA). Acetone (purity 99.5%) was purchased from Honeywell (Charlotte, NC, USA), and isopropanol (purity 99.5%) from ROTH (Karlsruhe, Germany).

### 2.2. Methods

#### 2.2.1. Preparation and Characterization of Hybrid Sensing Films

The gelatin hybrid sensors containing the ionic liquids (ILs) [BMIM][DCA] or [BMIM][Cl] and the liquid crystal 5CB were prepared as previously described [1,13]. To produce the sensing films with PDMS, the polymer solution was firstly prepared by adding the curing agent in a 1:10 ratio and leaving the mixture in the desiccator for 20 min. Then, the prepared PDMS solution was added to a heated glass vial containing the IL and the liquid crystal, and finally milliQ water was added. The final formulation presented two separated phases, which were manually stirred with a tip and finally collected and deposited on a clean glass slide. The spreading of the deposited material alongside the whole glass slide was conducted using a quadruplex with a predefined thickness of 30 µm (TQC Sheen) (Figure 1). For control sensors, the ionic liquid was not included in the formulation.

The films were left at room temperature and a controlled humidity (50% relative humidity) before further use. For the morphological characterization of the prepared films, we used polarized optical microscopy (POM) and scanning electron microscopy (SEM). For the POM analysis, images were taken under crossed (at 90°) and semi-crossed (at 45°) polarizers, as well as under the absence of polarizers (bright field) in a Zeiss Axio Observer. Z1/7 microscope equipped with an Axiocam 503 color camera and ZEN 2.3 software for image acquisition and processing. As for the scanning electron microscopy (SEM), observations were conducted on a Carl Zeiss AURIGA CrossBeam (FIB-SEM) workstation coupled with energy dispersive X-ray spectroscopy (EDS). The dried hybrid gel materials were previously coated with an Ir and Au/Pd conductive film to avoid charge effects. Stability to long-time storage was also evaluated by following a selected optically active region of interest by POM, for a period of a month.

#### 2.2.2. Response of Hybrid Gel Gas Sensors to Distinct Humidity Environments

Humidity reference sensors (HTU21D-F, Adafruit, New York, NY, USA, with an accuracy of ±2% RH and a response time between 5–10 s at 63% of the signal) were installed in the outlet of the detection chamber of the optical signal transducer device to record the variations of relative humidity in the chamber and in the cap of the saturated salt solution vial to record the generated RH (Figure S1). The measurements of the RH sensor in the saturated salt solution vial were synchronized with the measurement of the optical signal using an Arduino due, which controls the signal transducer device operation. To generate controlled levels of relative humidity, the sample delivery system depicted in Figure S1 was assembled and adapted to the optical e-nose device [11,13]. Two mass flow controllers (MFC1, MC-5SLPM-D/5M and MFC2, MC-2SLPM-D/5M, Alicat Scientific Inc., Tucson, AZ, USA) were fed with nitrogen as a carrier gas. A glass vial containing a supersaturated salt solution at room temperature (22 ± 2 °C) was installed at the outlet of the MFC2. Distinct RH levels were generated in the detection chambers by bubbling the nitrogen stream in supersaturated solutions of different salts [13] (Table S1) before entering the detection chamber.

Triplicates of sensing films were placed in the detection chamber and exposed to five humidification/drying periods for each RH level. The optical signals were acquired with the associated in-house developed optical signal transducer at constant temperature (room temperature of 22 ± 2 °C) [1,13,14,15,16]. To establish the humidification and drying periods, the MFC1 flow rate was constant at 1.5 slpm while MFC2 was programmed to alternately switch the flow rate between 120 s at 0 slpm (drying period) and 120 s at 1.5 slpm (humidification period). During the humidification period, the films were exposed to humidified nitrogen. During the drying period, dry nitrogen purged the detection chamber to ensure 0% RH. Independent experiments were made for each RH level, with three replicates of each sensing film type.

#### 2.2.3. Performance of PDMS and Gelatin Hybrid Gels as Sensors for VOC Discrimination

To assess the performance of the hybrid gels as sensors and to classify 12 distinct VOCs with small structural differences, a tailor-made optical e-nose was used [11,13]. The device contains a detection chamber with six sensor slots positioned between two crossed polarizers and employs two pumps that function alternately to expose the sensors either to VOCs, or to ambient air for recovery inside the chamber. The detection chamber is connected to a signal transduction unit that digitalizes the optical sensing signal and sends it to a computer, where it is presented in real time and stored for further processing. The acquisition of signals is possible because liquid crystal molecules act as optical probes owning to their birefringence, providing the measurable optical signals that are being analyzed. Due to the presence of the ionic liquid and its induced anchoring, liquid crystal molecules adopt a radial configuration, which exhibits typical optical textures observable under POM with crossed polarizers. This corresponds to the baseline level in the optical e-nose experiment—the baseline optical signal corresponds to the amount of polarized light being transmitted by the hybrid sensing film and reaching a photodetector in the transduction unit. However, when VOCs interact with hybrid gels, the liquid crystal molecules disorganize and gradually become less birefringent, which results in a significant decrease of the initially transmitted polarized light, and an increase of the optical signal from the baseline level. This process is reversible once the volatiles are desorbed from the gels, by pumping ambient air. Throughout the optical experiment, changes in light intensity are read by the photodetector, during both exposure and recovery cycles, so they can be used for subsequent analysis. This phenomenon is the basis for the optical VOC sensing experiment [1,13,14,15].

The sensors were positioned in the six sensor slots of the detection chamber, including three films of each PDMS and gelatin matrix gels—PDMS with [BMIM][DCA], PDMS with [BMIM][Cl], gelatin with [BMIM][DCA], gelatin with [BMIM][Cl] and the two control sensors (the formulation without ionic liquid). Their responses were tested against a set of 12 saturated atmospheres of VOCs: heptane, hexane, chloroform, toluene, dichloromethane, diethyl ether, ethyl acetate, acetonitrile, acetone, ethanol, methanol and acetic acid (VOCs listed in order of exposure). The solvents were kept in a thermostatized bath at 37 °C, for at least 15 min prior to exposure. The VOCs were pumped for 5 s into the detection chamber, followed by 15 s of ambient air for recovery (RH between 42–68%, temperature = 22 ± 2 °C), completing 1 cycle. A total of 45 cycles were performed, totalizing 15 min of duration per test. The optical signals of all sensing films were acquired at a sampling rate of 90 Hz, and assays were performed in triplicates. The signals were then analyzed using python programming tools previously developed, including an automatic classifier algorithm based on support vector machines [1,11,13,14,15,16,17].

#### 2.2.4. VOCs Limit of Detection Using PDMS and Gelatin Hybrid Gels as Sensors

To sample known concentrations of VOC (ethanol, acetone, toluene and hexane; Table S2) under controlled RH levels in the detection chamber, the apparatus depicted in Figure S2 was assembled. The VOC concentrations generated with this method were estimated as detailed in the Supporting Information. The dilution MFC (MC-5SLPM-D/5M, Alicat Scientific Inc.) was fed with nitrogen gas, which was bubbled through distilled water to generate an RH of 50% in the detection chamber or sent directly to the detection chamber to ensure an RH of 0%. The carrier MFC (MC-2SLPM-D/5M, Alicat Scientific Inc.) was also fed with nitrogen gas, which was bubbled through a known volume of pure solvent to generate a known flow rate of VOC vapor in the nitrogen carrier stream. The bubbler system is composed of a dip tube attached to a porous filter. The flow rate of VOC vapor and water vapor at the output of the bubbler flask were calculated as detailed in the Supplementary Information. The dilution MFC was set at a fixed flow rate (1.5 slpm, for ethanol, toluene and hexane or 5.0 slpm, for acetone). The carrier MFC was programmed to vary the flow rate in increments of 0.05 slpm (between 0.1 and 1.5 slpm) and repeat five times each flow rate step. In this way, five replicates of each VOC concentration were sampled to the detection chamber of the optical e-nose device during exposure/recovery cycles. To generate the exposure/recovery cycles, a two-way solenoid valve was used. The valve was programmed to alternately direct the nitrogen carrier stream (Figure S2) to the exposure or the recovery path using an automated temporized switching: (i) 5 s ON—Exposure; (ii) 15 s OFF—Recovery. During the exposure period, the nitrogen carrier stream, which bubbles through the solvent, is mixed with the dilution nitrogen stream (dry or humid) and, finally, introduced in the detection chamber. During the recovery period, the carrier stream is directed to an exhaust line and the detection chamber is purged with dry or humid nitrogen gas. The experiments were performed at a constant temperature (room temperature of 22 ± 2 °C).

A custom-made python script (Python 3.7, using Alicat library 0.2.2) was used to program the carrier MFC and solenoid valve operation, as well as to synchronize them with the optical e-nose device and the readings of the reference RH sensor at the outlet of the detection chamber [11].

#### 2.2.5. Signal Processing

The raw optical signals yielded by the hybrid sensors were firstly filtered with a median filter (to attenuate sporadic high intensity noise peaks) and smoothed with a Hanning window smoothing filter (to attenuate high frequency noise). Then, using a custom-made Python 3.7 script, the resulting signals were divided in cycles. The relative optical signal (*R_S_*) of each cycle was determined by subtracting its offset and normalizing it to its baseline, according to Equation (1):

(1) $$Relative\;optical\;signal\;\left(R_{S}\right)=\frac{signal-baseline}{baseline}$$

where *signal* is the smoothed and filtered cycle signal and *baseline* is the average of the signal immediately before (10 sample points) the start of the exposure period.

Regarding the humidity assays, for each cycle, the following features were extracted from the relative optical signal using custom-made Python 3.7. scripts: maximum derivative, minimum derivative and relative amplitude (*R_a_*), defined as the maximum variation of the relative optical signal (it can be either a maximum or a minimum, depending on the shape of the signal).

The minimum and maximum derivatives were used to calculate the slope of the signals in the humidification and drying periods, which correspond to the response speed to relative humidity step changes. The response time (*t_res_*), defined as the time required to reach the maximum variation of the relative optical signal (*R_S_*) after the start of a humidity step change (*t*_0_), was estimated from the response speed Equation (2) both for the humidification and for the drying periods of the experiments.

(2) $$response\;speed\;\left(s\right)=\frac{R_{S}}{t_{res}-t_{0}}$$

The relative amplitude of the signals in the presence of VOCs were corrected by subtracting the relative amplitudes of the signals to control tests without VOC, in order to eliminate the effect of RH changes and electrical noise inherent to the design of the MFC and e-nose system.

#### 2.2.6. Automatic VOC Discrimination

To assess the ability of each sensing material to identify VOCs based on the different shapes of the optical signals, these were first normalized, so their features could be extracted, and used as input variables to build an automatic classifier algorithm based on a Support Vector Machine (SVM), as reported previously [1,13,14,15,16]. For each sensor, the classifier was trained and tested regarding its performance to make predictions of the nature of the tested VOCs. The performance of the classifier was represented in the form of confusion matrices, which demonstrate the rate of correct and incorrect predictions given by the sensors, upon exposure to the volatiles. The VOC identification ability of each sensor was given by the rate of correct predictions for that VOC [1,13,14,15,16].

## 3. Results and Discussion

### 3.1. Gelatin and PDMS Hybrid Films

Liquid crystal molecules are appealing for sensing purposes due to their high molecular order and intrinsic stimuli-responsive nature, turning them into fast optical probes. In particular, the confinement of liquid crystal molecules into spherical arrangements increases the available surface area and yields a variety of optical textures that can be used as input signals [18,19]. The possibility to encapsulate ordered droplets of liquid crystal molecules within macroscopic matrices is an ideal approach for accurate sensing. In particular, the entrapment of liquid crystal droplets stabilized by ionic liquid molecules into gelatin ionogel matrices has proven suitable to yield robust thin films for gas and VOC-sensing [1,20]. To better understand the effect of the polymeric matrix on the sensing performance of these materials in humid conditions, both hydrophilic and hydrophobic polymeric matrices were selected for this study, while maintaining the ionic liquid ([BMIM][DCA] or [BMIM][Cl]) and liquid crystal (5CB) moieties constant. Type B gelatin from bovine skin (gelatin bovine) was selected as the hydrophilic matrix for comparison against the hydrophobic polydimethylsiloxane (PDMS) polymer (Figure 1). PDMS is an optically clear organosilicon polymer that has been explored in gas sensing as a matrix to encapsulate liquid crystal molecules (as in polymer-dispersed liquid crystals) [21,22] or as a hydrophobic coating to reduce humidity interference in gas sensors [10].

**Figure 2:**
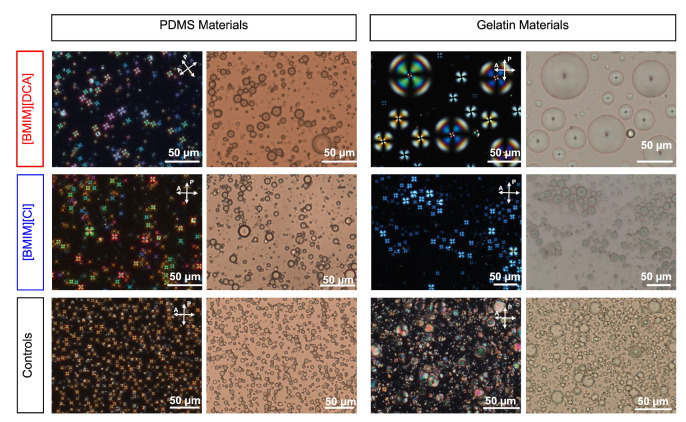
Examples of polarized optical microscopy (POM) images of optically active areas obtained for the sensing films under crossed polarizers (left columns) and bright field (right columns).

The produced gelatin and PDMS-based optical hybrid gels were firstly characterized by SEM and polarized optical microscopy (POM) to observe the morphological and optical textures, and to infer on liquid crystal molecular arrangements within the materials (Figure 2 and Figures S3 and S4).

In gelatin-based films, SEM images confirmed that the flexible, and yet robust, gelatin matrix contains droplets at its surface as previously reported [1]. For the PDMS-based films, a more uniform surface was observed, with no visible droplets, which indicates that these are likely placed inside the matrix or that the matrix was deteriorated during sample preparation (Figure S3).

Regarding the optical textures observed by the POM (see Figure 2), the PDMS control films (with no ionic liquid) exhibited almost monodispersed liquid crystal droplets, where the majority of them feature a radial configuration due to a perpendicular orientation of the liquid crystal molecules at the droplet interface [23]. This is evidenced by the Maltese cross pattern seen in POM with crossed polarizers. According to Bronnikov et al. [24,25], the semi-flexible nature of the PDMS matrix (due to the presence of the curing agent) coupled with the ratio of the liquid crystal:PDMS content used (1:10 *v*/*v*) allows for better phase separation between the liquid crystal and the polymeric matrix. This effect prevents droplet coalescence, leading to the formation of small radial droplets (on average of 10–15 µm diameter, Figure S4) [23,26,27,28]. On the other hand, this is not the case for the gelatin-based control formulation. Poor phase separation is observed between the liquid crystal and the gelatin matrix, which leads to droplet coalescence. Furthermore, since the liquid crystal:gelatin ratio is different (1:5 *v*/*v*) when compared to the PDMS control gel, polydisperse and occasionally irregularly shaped droplets are detected as a result. The droplets are very polydisperse in size and in most cases are significantly larger than those observed in the PDMS control gels. This induces weaker anchoring conditions between the liquid crystal and the polymer, leading to formations which feature a variety of configurations, typically bipolar [27].

Interestingly, the ionic liquid presence in hybrid gels leads to distinct optical textures and droplets sizes (Figure 2). For the PDMS-based hybrid gels, radial droplets are formed exhibiting on average a smaller diameter than the control gels, namely 5–10 µm (with [BMIM][DCA]) and 1–5 µm (with [BMIM][Cl]) (Figure S4). In the case of gelatin, the presence of ionic liquids alters the droplet size and morphology. Both [BMIM][DCA] and [BMIM][Cl] exhibit a surfactant-like effect that provides a stable perpendicular anchoring to the liquid crystal molecules, and also stabilizes the spherical shape of the droplets [1,12,13]. The formed liquid crystal droplets assume a radial profile with an average diameter of 15–20 µm in the case of [BMIM][DCA] and 5–10 µm in the case of [BMIM][Cl] (Figure S4). It should be noted that the presence of PDMS in hybrid gels restricts the growth of the nematic phase of the liquid crystal 5CB, resulting in smaller liquid crystal droplets when compared to gelatin-based hybrid gels. Additionally, it is evident that the ionic liquid anion influences droplet diameter. According to Esteves et al. [13], [DCA^−^] and [Cl^−^] anions give rise to different physical constraints upon interactions with the imidazolium headgroup [BMIM^+^]. Since both nitrile and chloride anions exhibit a similar ionic radius (191 and 181 pm, respectively) [29] and considering that [DCA^−^] contains two nitrile groups, it is likely that due to steric hindrance at the droplet interface, the [DCA^−^] anion facilitates the formation of larger droplets when compared to those containing [Cl^−^].

To evaluate the stability of the hybrid gels to storage at room temperature and controlled humidity conditions (50% RH), we monitored the films by POM during one-month. In general, droplets in [BMIM][DCA] materials, using either PDMS or gelatin, lead to more stable to storage optical sensors when compared to those bearing [BMIM][Cl] (Figure S5).

### 3.2. Optical Response of Sensing Films to Humidity

**Figure 3:**
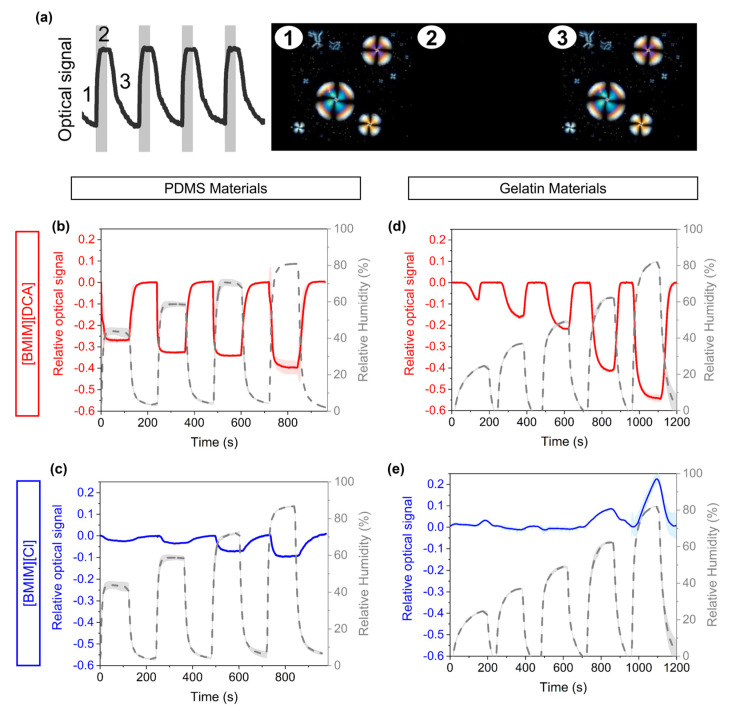
Relative optical signals of the sensing films to varying relative humidity. (**a**) Typical profile of the relative optical signals obtained during VOC-exposure and recovery cycles, as well as the corresponding optical morphological changes observed by POM in the hybrid gels during those cycles. (**b**) Signals yielded by a film composed of PDMS, [BMIM][DCA] and 5CB; (**c**) signals yielded by a film composed of PDMS, [BMIM][Cl] and 5CB; (**d**) signals yielded by a film composed of gelatin, [BMIM][DCA] and 5CB; (**e**) signals yielded by a film composed of gelatin, [BMIM][Cl] and 5CB. The plots illustrate representative examples of the signals of a single sensor exposed to different RH step changes.

The presence of liquid crystal molecules encapsulated within droplets in the PDMS and gelatin matrices makes it possible to study the changes in optical textures derived from the interaction of chemical analytes with the sensing films. In particular, the liquid crystal molecules inside the droplets can manipulate the polarization of light, allowing the transmission of light in optical devices under crossed polarizers. Based on previous works, the disruption in the organization of liquid crystal molecules within the droplet leads the molecular ordering to reversibly change from the nematic (see radial droplets in Figure 3a, points 1 and 3) to the isotropic phase (see point 2; dark image in Figure 3a) depending on the absorption and desorption of volatile analytes. Thus, the corresponding changes in the transmission of light work as a sensing signal for the adsorption and desorption of analytes. This optical signal is transduced by a custom-made e-nose device described in previous works [1,11,13,14,15,16,17], where the optical signal is inversely proportional to the light transmitted through the sensing film.

**Figure 4:**
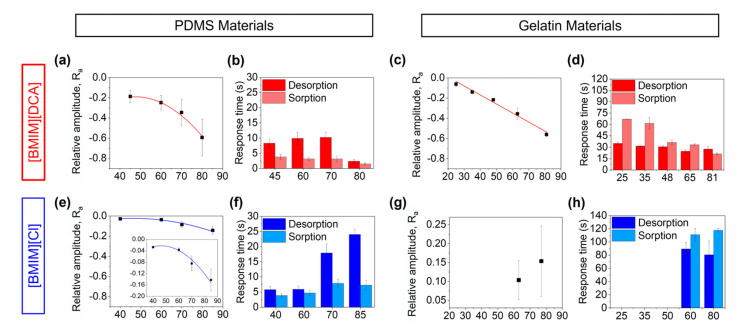
Analysis of the relative amplitude and response time to humidity of hybrid films made with PDMS or gelatin and the ionic liquids [BMIM][DCA] (**a**–**d**) or [BMIM][Cl] (**e**–**h**). (**a**–**h**) Response time of the optical signal of hybrid films as a function of %RH, representing the required time to reach a stable-state response to humidification from 0% to different RH levels (sorption) and to drying from those RH levels to 0% RH (desorption). (**a**) Polynomial fit of the variations of the relative amplitude of the optical response of [BMIM][DCA]-based hybrid films made with PDMS as a function of %RH (optical signal relative amplitude = −0.0004 × %RH^2^ + 0.0421 × %RH−1.2208, R^2^ = 0.99). (**c**) Linear fit of the variations of the relative amplitude of the optical response of [BMIM][DCA]-based hybrid films made with gelatin as a function of %RH (optical signal relative amplitude = −0.009 × %RH + 0.188, R^2^ = 0.98). (**e**) Polynomial fit of the variations of the relative amplitude of the optical response of [BMIM][Cl]-based hybrid films made with PDMS as a function of %RH (optical signal relative amplitude = −7 × 10 − 5 × %RH2 + 0.0057 × %RH − 0.1475, R^2^ = 0.98). (*n* = 3 independent replicates of each hybrid film were used to measure the optical responses to humidity).

To study the effect of humidity on the optical signal of the hybrid gel thin films, the e-nose device was coupled to a humidity delivery setup (Figure S1). The hybrid thin films were exposed to alternated cycles of dry (0% RH) and humid nitrogen (between 25 and 85% RH) (Table S1) and the raw optical signals (Figure S6) were recorded. The relative optical response to increasingly higher levels of relative humidity is represented in Figure 3 for the hybrid thin films and further analyzed in Figure 4. The control films (without ionic liquid) are nearly unaffected by humidity, yielding raw optical signals with extremely low amplitudes, even when exposed to the largest RH variation (0–80%) (Figure S6c,f).

Considering now the hybrid films, the sensors’ response to humidity is mostly governed by the nature of the ionic liquid used. The sorption and desorption of water molecules has a similar effect on the optical signal of films containing [BMIM][DCA] in PDMS and gelatin (Figure 3b,d), as in general the reduction of humidity content leads to a reduction on the amount of light transmitted (i.e., an increase in the relative optical signal). In [BMIM][DCA]-gelatin materials, the drying of the films is associated with a liquid crystal transition to a complete isotropic state, visualized by the transition of the optical signal to the maximum scale voltage (Figure S6d). The relative optical response varies linearly with the %RH step variation and response times vary between 20–60 s (responses are faster as %RH increases) (Figure 4c,d). For [BMIM][DCA]-PDMS materials, the raw optical signal does not fully transition to the maximum scale voltage when dried (Figure S6a) (the raw optical signal amplitude is lower than for gelatin films), indicating that the liquid crystal is kept in a nematic phase although with a reduction on the total amount of light transmitted, as seen by the variation of the relative optical signal shown in Figure 3b. The variation of the relative optical response as a function of %RH step change follows a polynomial fit and response times vary between 1–10 s (Figure 4a,b).

Considering now the hybrid gels containing [BMIM][Cl], it is observed that the amplitude of the relative optical signal is very low when compared to the same hybrid gels but with [BMIM][DCA] (Figure 3c,e). This phenomenon is also evident when comparing the raw signals (Figure S6b,e). This is mostly attributed to the fact that [BMIM][Cl] holds strongly onto water, and as such the films are not so altered by variations in relative humidity as the anion Cl^−^ is already equilibrated with water molecules [11]. As shown in Figure S6b,e, for PDMS-based gels the increase of humidity leads to a decrease in the optical signal (i.e., an increase in transmitted light), whereas for gelatin-based gels the increase of humidity leads to an increase in the optical signal (i.e., a decrease in transmitted light). This difference in behavior is identical to that observed for the control films (Figure S6c,f) and is thus attributed to the matrix properties, namely to LC droplets movement in gelatin-based films due to fluidification of the matrix, as we observed in our past work [11]. In PDMS-based signals, fluidification is not expected due to the polymer hydrophobicity. As such, different phenomena should explain the slight increase in transmitted light. Overall, our results indicate that in the absence of ionic liquid in the sensors composition (control films), the influence of humidity in the optical sensors’ response is governed by the hydrophobic properties of the polymeric matrix. For PDMS control sensors, the higher hydrophobicity of the matrix repels water molecules and therefore the liquid crystal molecules are barely affected by the presence of water molecules. When adding the ionic liquid into the composition, the measured optical signal is mostly affected by the nature of the anion from the ionic liquid, with a higher humidity effect for [BMIM][DCA]-containing hybrid gels. Still, the amplitude of the optical response for PDMS-based hybrid gels is lower than for gelatin-based gels, indicating a lower water interference.

### 3.3. Hybrid Gels Sensors to Discriminate between Distinct VOCs

**Figure 5:**
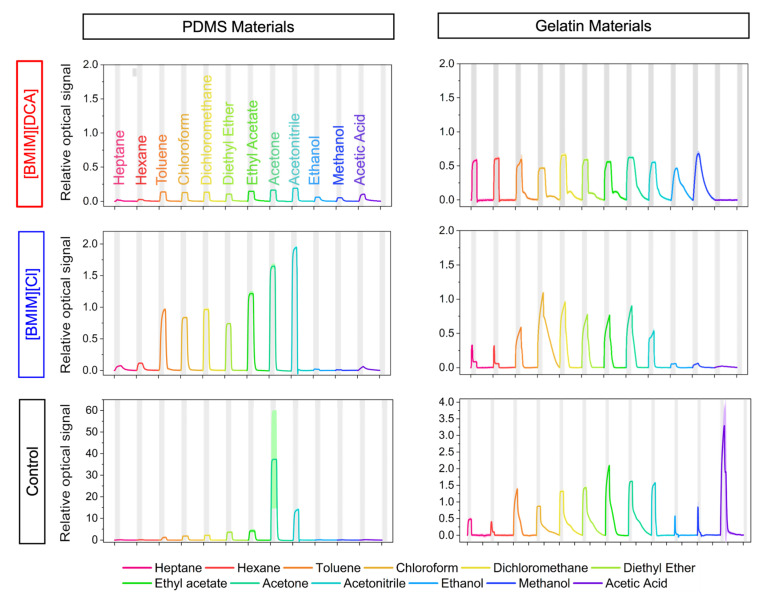
Representative signals of the optical amplitude responses of all hybrid gels and control gels studied. The typical profile of the signals obtained during the exposure and recovery cycles in the optical e-nose. The plots were generated by python.

After studying how hybrid gels based on PDMS and gelatin behaved to the presence of humidity, we set to understand how these materials performed as VOC sensors in ambient humid environments (room temperature and RH between 40–60%). The hybrid gels and respective controls were sequentially exposed to twelve VOCs from distinct chemical classes and small structural differences. The sensors were exposed to saturated atmospheres of the following compounds: heptane, hexane, chloroform, toluene, dichloromethane, diethyl ether, ethyl acetate, acetonitrile, acetone, ethanol, methanol and acetic acid (VOCs listed in order of exposure). The representative relative optical signals obtained for each sensor and VOC are shown in Figure 5.

Considering the [BMIM][DCA]-based sensors, gelatin materials yielded signals with higher relative amplitudes and distinct responses to VOCs when compared to PDMS. For example, gelatin materials were unresponsive to acetic acid but more responsive to hexane and heptane. For the [BMIM][Cl]-based sensors, those containing PDMS yielded relative signals with higher amplitude and sharper signals with fast response and recovery. In the case of the control sensors, the PDMS controls gave very low signals to VOCs except for acetone which yielded a relative optical signal 10 times higher than the IL-containing sensors. In the case of gelatin controls, relative signal amplitudes were also higher than the IL-containing sensors, in particular the response to acetic acid which was extremely high for IL-gelatin sensors. The signals’ response times depend on a combination of factors related to the composition of the sensing material (such as the hydrophilicity of the polymer matrix and the size of the droplets) and with the chemical nature of the VOC itself [1]. In fact, the sensors’ response profiles are likely influenced by the chemical interactions established between a given VOC and the polymer matrix. As the potential chemical interactions contribute to a stronger or weaker adsorption of VOC molecules to the polymeric matrix, different recovery profiles and recovery times are observed for the two types of sensors (Figure 5). Unlike PDMS, gelatin is composed by several amino acids that can interact with some of the tested VOCs [11] and favor their adsorption to the matrix, which slows down the recovery of gelatin-based sensors compared to PDMS-based sensors. That is the case of the responses to acetone, for example. On the other hand, apolar VOCs, such as hexane and heptane, are associated with a faster recovery in gelatin sensors and a slower recovery in PDMS sensors. In addition, larger droplets are slower to recover the radial configuration after the removal of the VOC than smaller droplets. Thus, a larger average size of the droplets found in gelatin-based sensors (Figure 2) likely also contributes to the observed delay in the signals’ recovery times.

**Figure 6:**
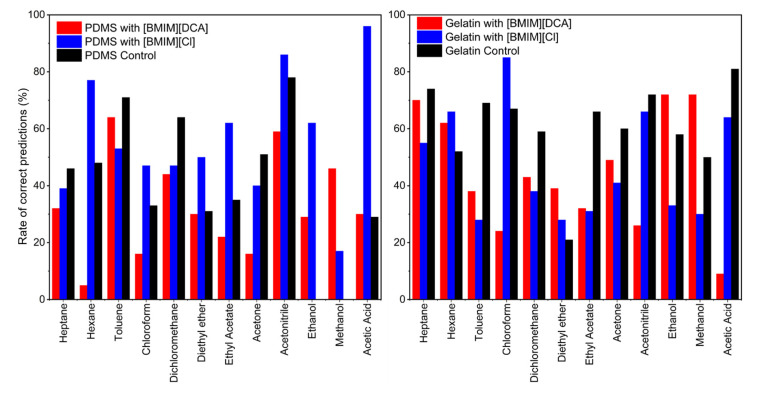
Average rates of correct prediction retrieved from the confusion matrices analysis regarding the ability of each sensor to predict each volatile being tested, during the optical e-nose experiments. A rate above 50% is considered a good classification performance of the volatile from the sensor. Sensors containing PDMS with [BMIM][DCA], [BMIM][Cl] and without ionic liquid (control), as well as sensors containing gelatin with [BMIM][DCA], [BMIM][Cl] and without ionic liquid (control) are represented.

After signals collection, the data were analyzed through machine learning tools, using several features and parameters of curve fitting models as inputs for support vector machine-based classification algorithm. This resulted in plots summarizing mean normalized wave signals deriving from each VOC–gel interaction yielding typical signals, and also confusion matrices (Figure S7), where the prediction performance of the classifier towards the VOC being tested is evaluated. A summarized plot of the classification performance results is presented in Figure 6. The bars represent the percentage of correct predictions for each VOC. Above 50% is considered a good classification of the volatile from the sensor. In general, the [BMIM][DCA]-based sensors yield classification systems lower than those obtained for [BMIM][Cl]-based sensors.

The volatiles prediction performance, in general, was higher for the PDMS with [BMIM][Cl] ionic liquid, achieving, on average, 56% of correct predictions, being the best results for acetic acid (96%), acetonitrile (86%) and hexane (77%), and the worst for methanol (17%). On the other hand, the lowest rates of correct predictions were obtained for the same matrix using the [BMIM][DCA] ionic liquid, which yielded, on average, a correct prediction rate of 33% scoring relatively good values for acetonitrile (59%) and toluene (64%).

The [BMIM][DCA]–gelatin matrices presented better scores for ethanol (72%), methanol (72%), heptane (70%) and hexane (62%) prediction, when compared to [BMIM][DCA] in PDMS, obtaining an average rate of correct predictions of 45%. For [BMIM][Cl] in the gelatin matrix, good scores were also found in general for volatiles such as acetic acid (64%), acetonitrile (66%) and hexane (66%), similar to [BMIM][Cl] in the PDMS matrix, which points to the critical role of ionic liquid in VOCs discrimination. Additionally, [BMIM][Cl] in gelatin presented the best discrimination for chloroform (85%) of all gels.

The presence of PDMS in the formulation of the sensing films favored the distinction of both toluene and acetonitrile, while the gelatin matrices performed better for heptane, hexane and chloroform discrimination. Overall, the results corroborate the joint effect of all the moieties of the multicomponent hybrid gels during VOC interaction, and also point out the important impact of the IL on VOCs discrimination. Furthermore, the diversity in the sensors’ composition and the complementary accuracy results obtained open the possibility to combine the results from several sensors to improve VOC discrimination.

### 3.4. VOCs Limit of Detection Using PDMS and Gelatin Hybrid Gels as Sensors

After assessing the effect of humidity and the potential for VOC sensing in humid conditions using PDMS and gelatin hybrid gels, we assessed their limit of detection for selected VOCs under humid conditions. Ethanol, acetone, toluene and hexane were chosen as the representatives of chemical groups with distinct polarity, hydrophilicity and affinity to the components of the studied materials [1,30]. The optical e-nose device employed in the previous section was coupled to a VOC delivery system to monitor the film’s optical responses upon exposure to increasing concentrations of VOCs diluted in humid (50% RH) nitrogen. VOC concentrations were controlled by manipulating the samples’ temperature and the flow rates of the carrier and dilution nitrogen streams in the VOC delivery system. To generate the humid stream, a water bubbling system was installed in the dilution stream (Figure S2).

To ensure a constant baseline for the VOC experiments, the films were first allowed to stabilize for 15 min in 50% RH. In addition, a calibration blank assay under the same humidity and nitrogen flow rates but without VOC was performed, to discount the interference of noise in the signal, where it was previously seen that the optical signal from noise causes a minimal interference [11].

**Figure 7:**
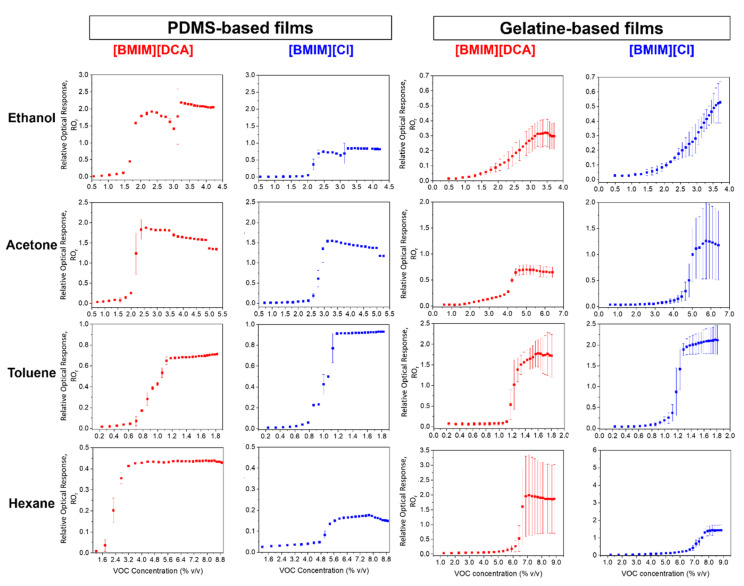
Variation of the relative optical response of hybrid films with the increase in concentration of ethanol, acetone, toluene and hexane, dissolved in humid (50% RH) nitrogen (*n* = 5 exposure cycles to VOC for each concentration).

The relative optical responses to increasing concentrations of VOCs under a controlled RH of the hybrid films containing [BMIM][DCA] or [BMIM][Cl] in a PDMS or gelatin matrix are represented in Figure 7 and Figure S8 shows the corresponding raw optical signals. The estimated limit of detection (LOD), the triggering concentration and the saturated response can also be found in SI (Tables S3 and S4). The triggering concentration is understood as the concentration of VOC molecules that is enough to reach the droplets’ surface and trigger a heightened phase transition of the LC droplets. Below this concentration, the variation in the relative optical response is minimal. Above, the variation is significantly larger until it reaches the saturated response.

The polarity of the VOCs influences the optical response of the films, as reported in our previous works [1,13,20]. More polar VOCs, such as ethanol and acetone, cause a baseline drift on the signal throughout time (Figure S8), while the less polar VOCs, such as toluene and hexane, provide a more stable and reproducible optical response (Figure S8). Non-polar VOCs tend to interact directly with the LC component of the hybrid films, thus conserving the configuration of the ionic liquid droplets, while polar VOCs likely interact mostly with the amphiphilic ionic liquids and, in the case of gelatin, with the polymeric matrix. Therefore, over time polar VOCs tend to alter the initial morphology of the IL-LC droplets, which could explain the drift in the baseline of the optical signal (Figure S8). Changing the anion of the IL from [DCA^−^] to [Cl^−^] does not cause an evident alteration in terms of VOC-sensitivity performance. The optical response of the films has very similar waveforms, but the limits of detection (LOD) towards each VOC differ for PDMS and gelatin-based films.

In both PDMS and gelatin-based films, the optical response to ethanol of films containing the [Cl]^−^ counterion is slightly weaker than the [DCA]-counterpart, likely because ethanol being very polar interacts strongly with surrounding water molecules, and films containing [BMIM][Cl] are less responsive to humidity than [BMIM][DCA]-based films. Additionally, the LOD in PDMS and gelatin-based films for [BMIM][DCA] is slightly lower than for [BMIM][Cl] (Tables S3 and S4).

It is also noted for ethanol that gelatin films do not exhibit a triggering concentration. It is thought that in gelatin-based films, ethanol interacts preferentially with the matrix, the water and the anion of the IL [11]. In PDMS films, ethanol would interact preferentially with the droplets, because with the matrix being hydrophobic [31], the only component to interact with ethanol is the amphiphilic IL; thus, the response mechanism functions similar to other VOCs (i.e., there is a triggering concentration that escalates the optical response).

For the remaining VOCs, it is observed that the triggering concentrations are lower for PDMS films than for gelatin (Tables S3 and S4). In gelatin films, the VOCs tend to perform H-bond and π-π interactions with the matrix [11]. PDMS is less prone to these interactions due to its chemical structure [25]; instead, VOCs penetrate the film at voids, and because PDMS is cross-linked, added to the lower polarity of the VOCs, the rate of diffusion is also affected [31]. Thus, the VOCs may preferentially interact with the droplets at the surface of the film [11].

The response to hexane is significantly different between PDMS and gelatin-based films. In addition, the LODs vary by changing the counter-ion of the IL (Tables S3 and S4). The detection limit is not detectable for [BMIM][DCA], and in [BMIM][Cl], PDMS-based films have a lower LOD (1.76% *v*/*v*) than what is observed for gelatin (2.27% *v*/*v*).

The more hydrophobic PDMS polymer easily interacts with the less polar VOCs, while gelatin likely has a greater affinity towards more polar VOCs. In the case of hexane, perhaps due to its greater hydrophobicity, it probably penetrates the IL droplets to interact with the LC and hydrophobic moiety of the IL, while the other VOCs are able to interact with the surface of the droplets [11].

In summary, the change in the polymeric matrix between the PDMS and gelatin of the hybrid films does not produce an evident improvement in VOC-sensitivity sensing-performance. Yet, due to the opposing nature of the two polymers, the films complement well one another, in terms of sensitivity to distinct VOCs.

## 4. Conclusions

Hybrid gel sensors were successfully produced using a PDMS matrix with 5CB liquid crystal and two different ionic liquids ([BMIM][Cl] and [BMIM][DCA]) and tested in an in-house built optical electronic nose device against a set of 12 different volatile organic compounds.

The presence of PDMS in the formulation of the sensing films seems to favor in general the distinction of both toluene and acetonitrile volatiles in general, as opposed to the presence of gelatin, which seems to do the same for heptane, hexane and chloroform instead. In this work, however, we conclude that the classification of VOCs through these experiments are a consequence of the interactions with the entire multicomponent system, and not just dependent on the matrix individual component, but also that choice of the IL seems to be key to achieve different VOCs discrimination.

The best overall VOC prediction performance was obtained for the PDMS with [BMIM][Cl] ionic liquid, yielding, on average a 56% rate of correct predictions, followed by PDMS control (scoring 41%), and the least accurate formulation was the PDMS with [BMIM][DCA] ionic liquid, achieving only 33% of general accuracy predicting volatiles.

When comparing the PDMS gels with the traditional gelatin sensing films with two different ionic liquids, in terms of morphology they diverge a lot in both droplets’ visual appearance and quantity. As for the stability to long-time storage, sensing materials containing [BMIM][DCA] (especially PDMS), proved to be the most stable gels, exhibiting radial stable droplets for over a one month period against the ones where [BMIM][Cl] ionic liquid is used, which proved to be very unstable (especially the gelatin films).

Studies regarding the optical response of the sensing films to different humidity levels were also conducted, and the results indicate that, overall, in the absence of ionic liquid in the sensors composition (control films), the influence of humidity in the optical sensors response derives essentially from the hydrophobic properties of the polymeric matrix.

In summary, the use of PDMS as a supporting matrix for the thin films changes the effect of humidity on the sensing performance compared to gelatin, but it does not entirely optimize it; due to the presence of the amphiphilic IL, when adding the IL into the composition, the measured optical signal is mostly affected by the nature of the anion from the IL. A higher humidity effect is clearly observed for [BMIM][DCA]-containing hybrid gels. The amplitude of the optical response for PDMS-based hybrid gels is lower than for gelatin-based gels, indicating a lower water interference. Generally, sensors containing [BMIM][DCA] are more responsive to humidity variations, while films containing [BMIM][Cl] remain very unresponsive in comparison.

VOC sensing-performance was also explored in this work, and conclusions can be drawn that neither gelatin nor PDMS-based films outperform one another, but the two distinct film recipes complement well each other. In the future, the sensors could be used in combination for VOC-sensing in distinct humidity conditions. Characterization of the sensors tackling issues such as the impact of temperature and the 3S (Sensitivity, Stability and Selectivity) should be studied in more detail to fully understand these new materials.

## Data Availability

Data supporting the reported results can be requested by directly contacting the authors.

## Supplementary Materials

The following supporting information can be downloaded at: https://www.mdpi.com/1424-8220/23/7/3531/s1, Table S1: Examples of supersaturated salt solutions and corresponding maximum relative humidity (RH) levels measured at ~20 °C at the outlet of the detection chamber. Distilled water generates the maximum relative humidity. Table S2: Estimated VOC concentrations to which gelatin and PDMS-based sensing films containing either the ionic liquids [BMIM][DCA] or [BMIM][Cl] were exposed. Table S3: Optical sensing performance of [BMIM][DCA] hybrid films of PDMS or gelatin at 50% RH. Table S4: Optical sensing performance of [BMIM][Cl] hybrid films of PDMS or gelatin at 50% RH. Figure S1: Experimental setup for humidity delivery coupled with the signal acquisition device. The sensors in the detection chamber are alternately exposed to dry (0% RH) and humid nitrogen (between 25–85% RH), by sampling nitrogen directly to the detection chamber or by bubbling nitrogen through supersaturated salt solutions at ambient temperature, respectively. Figure S2: Experimental setup for delivery of known VOC concentrations under dry and humid conditions coupled with the signal acquisition device, for the evaluation of PDMS-based films containing [BMIM][DCA] or [BMIM][Cl] ILs as gas sensitive optical sensors. Distinct VOC concentrations in dry and humid nitrogen are generated and sent to the detection chamber by manipulating the temperature of sample in the thermal bath and the flow rates of the dilution and carrier mass flow controllers (MFC). Figure S3: Scanning electron microscopy (SEM) images of Au/Pd coated films. (a) PDMS matrix with liquid crystal 5CB and ionic liquid [BMIM][DCA]; (b) PDMS matrix with 5CB and [BMIM][Cl]; (c) PDMS matrix with 5CB (control); (d) gelatin matrix with 5CB and [BMIM][DCA]; (e) gelatin matrix with 5CB and [BMIM][Cl]; (f) gelatin matrix with 5CB (control). Images were obtained by a Carl Zeiss AURIGA CrossBeam (FIB-SEM). Figure S4: Histogram of droplets diameter distribution for the films produced with PDMS or gelatin, 5CB liquid crystal and both ionic liquids [BMIM][DCA] and [BMIM][Cl], as well as PDMS control. Figure S5: POM images of constant optically active areas obtained for the PDMS and gelatin sensing films produced from day 1 till 1 month after production. Images were obtained by Zeiss Polarized Optical Microscope (Zen 2.3 software), with 90° polarizers at 10× magnification. Figure S6: Raw optical signals of sensing films to a step change of relative humidity from 0% to 80%. (a) hybrid film composed by PDMS, [BMIM][DCA], 5CB; (b) hybrid film composed by PDMS, [BMIM][Cl], 5CB; (c) PDMS control film (without IL); (d) hybrid film composed by gelatin, [BMIM][DCA], 5CB; (e) hybrid films composed by gelatin, [BMIM][Cl], 5CB; (f) gelatin control (without IL) film. Figure S7: Optical e-nose confusion matrices obtained, illustrating the prediction results regarding 12 VOCs based on the signals obtained from the hybrid gel sensors with both PDMS and gelatin matrix hybrid sensors with two different ionic liquids ([BMIM][DCA] and [BMIM][Cl]). The green squares in the diagonal represent the correct predictions made by the classifier, and the lighter blue squares outside of the diagonal represent the incorrect prediction of a VOC. Figure S8: Variation of the amplitude of the optical response of hybrid films with the increase in concentration (indicated in %(*v*/*v*) for each plot), of ethanol, acetone, toluene and hexane dissolved in humid (50% RH) nitrogen. (a), (b), (c), (d) Response amplitude of hybrid films composed by PDMS, the ionic liquid [BMIM][DCA] or [BMIM][Cl], 5CB and water. (e), (f), (g), (h) Response amplitude of hybrid films composed by gelatin, the ionic liquid [BMIM][DCA] or [BMIM][Cl], 5CB and water. (*n* = 2 exposure cycles to VOC for each concentration).
