# Correction: Oliveira et al. Effect of Polymer Hydrophobicity in the Performance of Hybrid Gel Gas Sensors for E-Noses. *Sensors* 2023, *23*, 3531

**DOI:** 10.3390/s24165144

**Published:** 2024-08-09

**Authors:** Ana Rita Oliveira, Henrique M. A. Costa, Efthymia Ramou, Susana I. C. J. Palma, Ana Cecília A. Roque

**Affiliations:** 1 Associate Laboratory i4HB—Institute for Health and Bioeconomy, School of Science and Technology, NOVA University Lisbon, 2829-516 Caparica, Portugal; 2 UCIBIO—Applied Molecular Biosciences Unit, Department of Chemistry, School of Science and Technology, NOVA University Lisbon, 2829-516 Caparica, Portugal

In the published publication [1], the first two authors should be marked as equal authors:

 

**Ana Rita Oliveira ^1,2,†^, Henrique M. A. Costa ^1,2,†^,**

^†^ These authors contributed equally to this work.

The authors state that the scientific conclusions are unaffected. This correction was approved by the Academic Editor. The original publication has also been updated.
