# Supplementary material for: Effect of Polymer Hydrophobicity in the Performance of Hybrid Gel Gas Sensors for E-Noses
Source: Sensors (Basel). 2023 Mar 28;23(7):3531. doi: 10.3390/s23073531 (PMC10098550; doi:10.3390/s23073531)
Supplement: Supplementary file 1 [file sensors-23-03531-s001.zip › sensors-2279347-supplementary.pdf]

sensors

Effect of Polymer Hydrophobicity in the Performance of Hybrid Gel Gas Sensors for E-Noses

Ana Rita Oliveira [1,2], Henrique M. A. Costa [1,2], Efthymia Ramou [1,2], Susana I. C. J. Palma [1,2] and Ana Cecília A. Roque [1,2,*]

[1] Associate Laboratory i4HB—Institute for Health and Bioeconomy, School of Science and Technology, NOVA University Lisbon, 2829-516 Caparica, Portugal
[2] UCIBIO—Applied Molecular Biosciences Unit, Department of Chemistry, School of Science and Technology, NOVA University Lisbon, 2829-516 Caparica, Portugal
[*] Correspondence: cecilia.roque@fct.unl.pt

Supplementary Figures and Tables

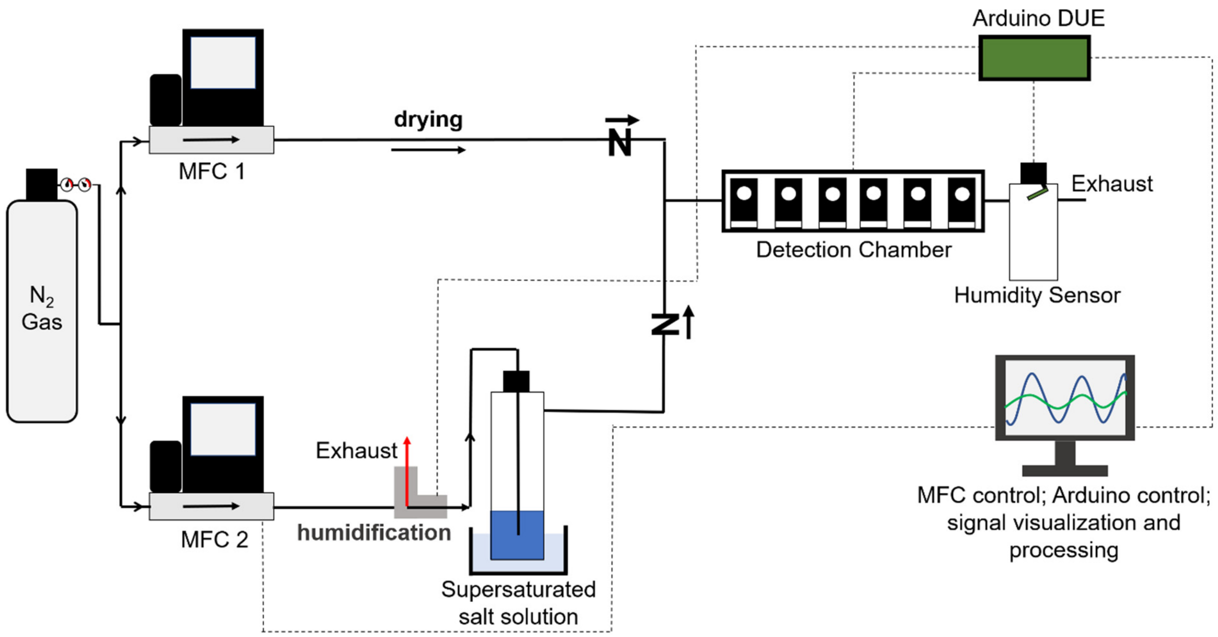

Figure S1. Experimental setup for humidity delivery coupled with the signal acquisition device. The sensors in the detection chamber are alternately exposed to dry (0% RH) and humid nitrogen (between 25-85% RH), by sampling nitrogen directly to the detection chamber or by bubbling nitrogen through supersaturated salt solutions at ambient temperature, respectively.

Table S1. Examples of supersaturated salt solutions and corresponding maximum relative humidity (RH) levels measured at ~20º C at the outlet of the detection chamber. Distilled water generates the maximum relative humidity.

Supersaturated salt solution	Maximum RH at outlet of the detection chamber (%)
Magnesium Chloride	45
Sodium Bromide	60
Sodium Chloride	70
Distilled Water	85

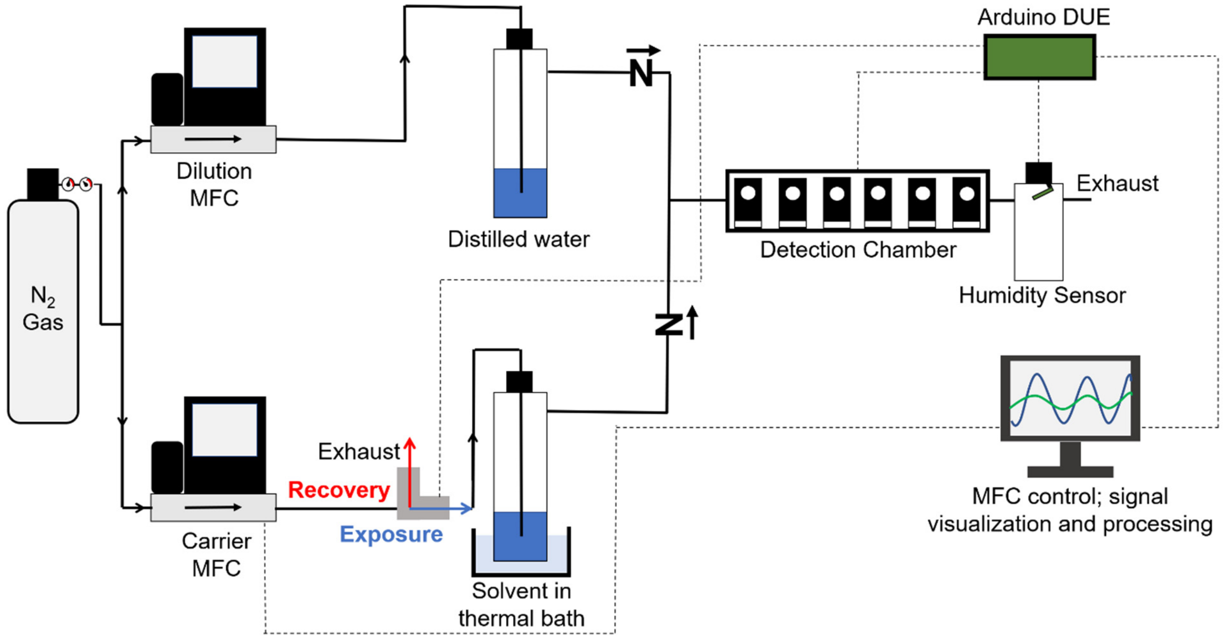

Figure S2. Experimental setup for delivery of known VOC concentrations under dry and humid conditions coupled with the signal acquisition device, for the evaluation of PDMS-based films containing [BMIM][DCA] or [BMIM][Cl] ILs as gas sensitive optical sensors. Distinct VOC concentrations in dry and humid nitrogen are generated and sent to the detection chamber by manipulating the temperature of sample in the thermal bath and the flow rates of the dilution and carrier mass flow controllers (MFC).

The flow rate of VOC vapor and water vapor at the output of the bubbler flask, Fn, was estimated using the bubbler equation (Equation (S1)):

$$F_n = \frac{P_n^*}{P_i} \times F_c, \; n = v\,(VOC);\; w\,(water) \qquad \text{(S1)}$$

where P*n is the saturated vapor pressure of the solvent or of water at a given temperature, calculated by the Antoine Equation, or corresponding to the registered RH, respectively; Pi is the pressure of the inlet stream, given by the carrier MFC; and Fc is the flow rate of the nitrogen carrier stream.

Different VOC concentrations in the detection chamber were generated by varying the temperature of the solvent (20º - 28º C) and by mixing the carrier flow rates (nitrogen flow rate (Fc) and VOC flow rate (Fv)) generated at the output of the bubbler flask with the dilution flow rates (nitrogen flow rate (Fd) and water vapor flow rate, if present (Fw)) at different ratios. The resulting VOC concentration, in %(v/v), (Table S2) were estimated from Fv and the total mixed flow rates (Equation (S2)).

$$C_{VOC}\,(\%\,(v/v)) = \frac{F_v \times 10^6}{F_c+F_v+F_d+F_w}\,(ppm) \times 10^{-4}\left(\frac{\%(v/v)}{ppm}\right) \qquad \text{(S2)}$$

Table S2. Estimated VOC concentrations to which gelatin and PDMS-based sensing films containing either the ionic liquids [BMIM][DCA] or [BMIM][Cl] were exposed.

VOC	Range of concentrations sampled to the detection chamber (% (v/v))
Ethanol	0.50 – 4.30
Acetone	0.50 – 5.00
Hexane	1.20 – 9.00
Toluene	0.20 – 1.90

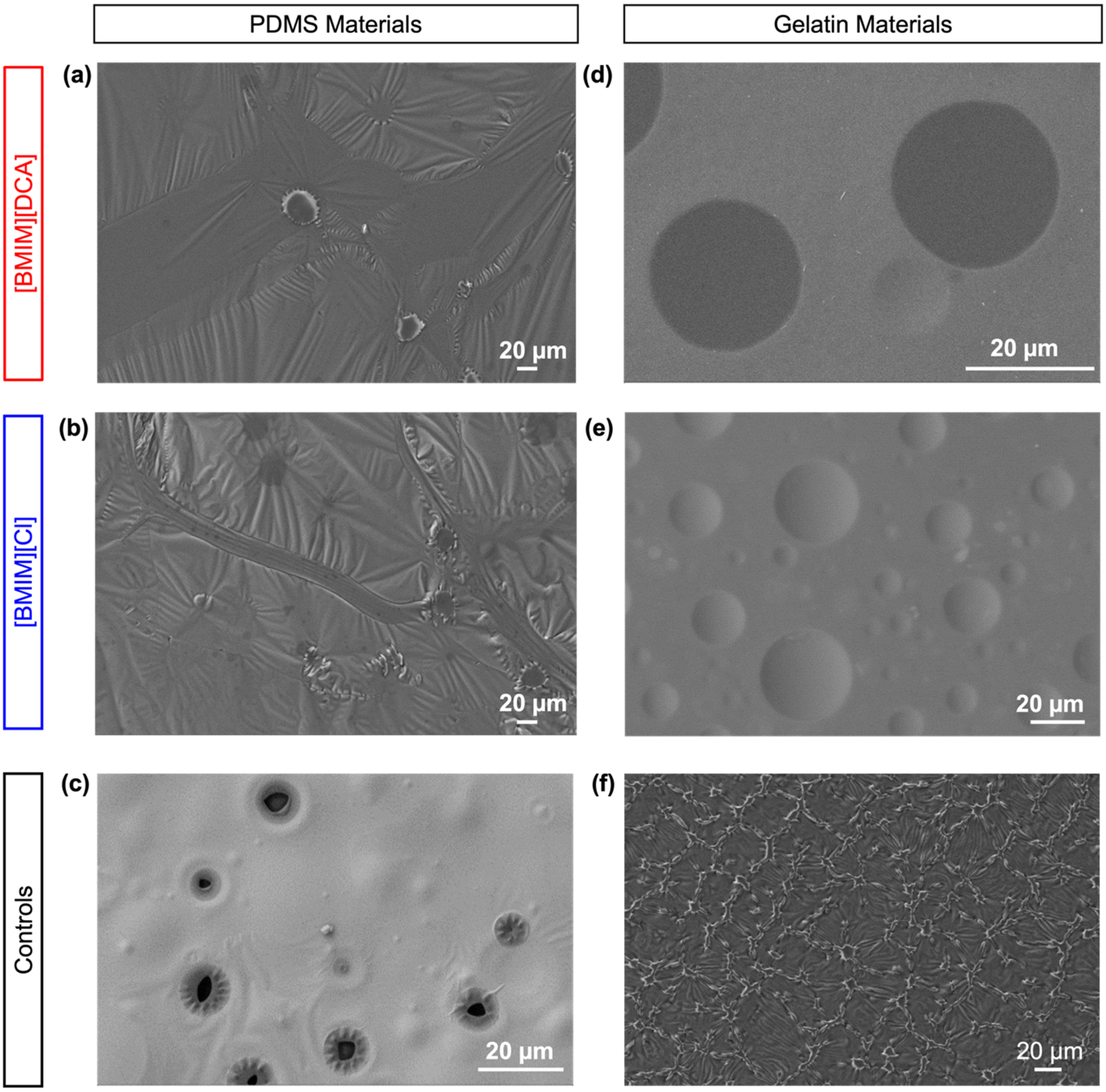

Figure S3. Scanning electron microscopy (SEM) images of Au/Pd coated films. **(a)** PDMS matrix with liquid crystal 5CB and ionic liquid [BMIM][DCA]; **(b)** PDMS matrix with 5CB and [BMIM][Cl]; **(c)** PDMS matrix with 5CB (control); **(d)** gelatin matrix with 5CB and [BMIM][DCA]; **(e)** gelatin matrix with 5CB and [BMIM][Cl]; **(f)** gelatin matrix with 5CB (control). Images were obtained by a Carl Zeiss AURIGA CrossBeam (FIB-SEM).

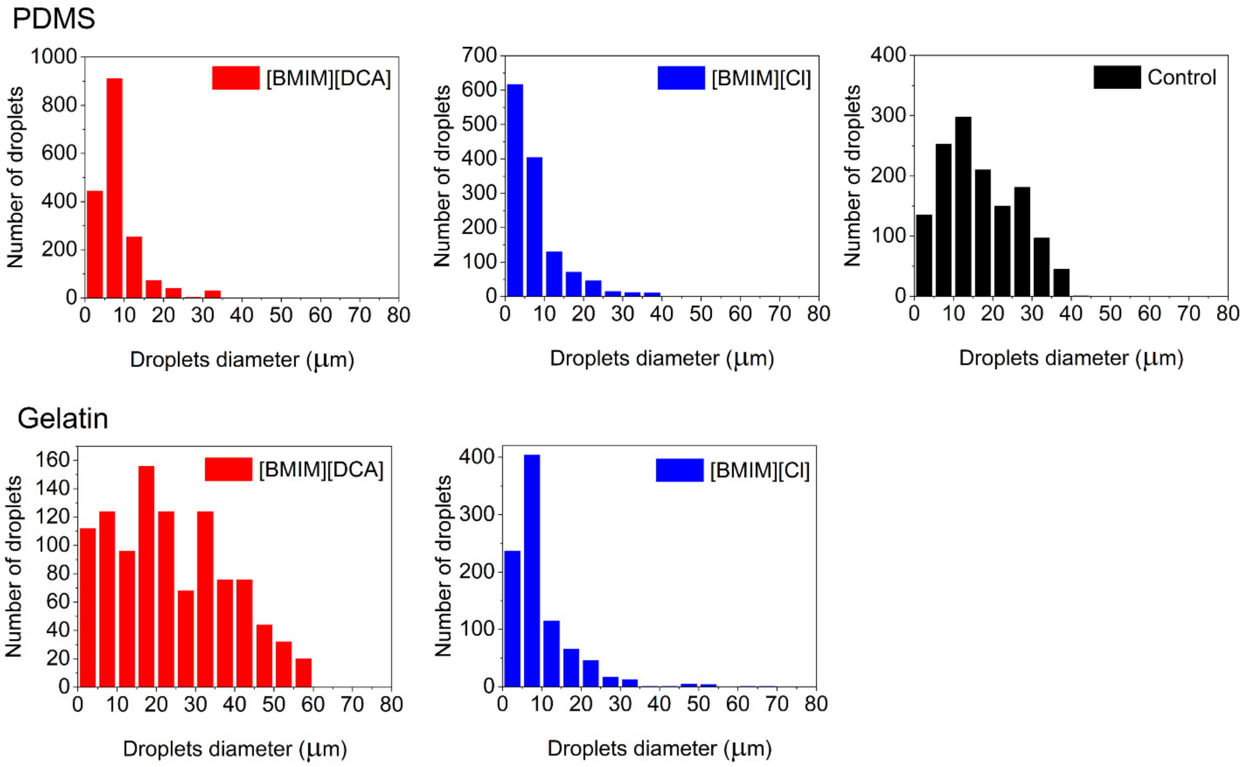

Figure S4. Histogram of droplets diameter distribution for the films produced with PDMS or gelatin, 5CB liquid crystal and both ionic liquids [BMIM][DCA] and [BMIM][Cl], as well as PDMS control.

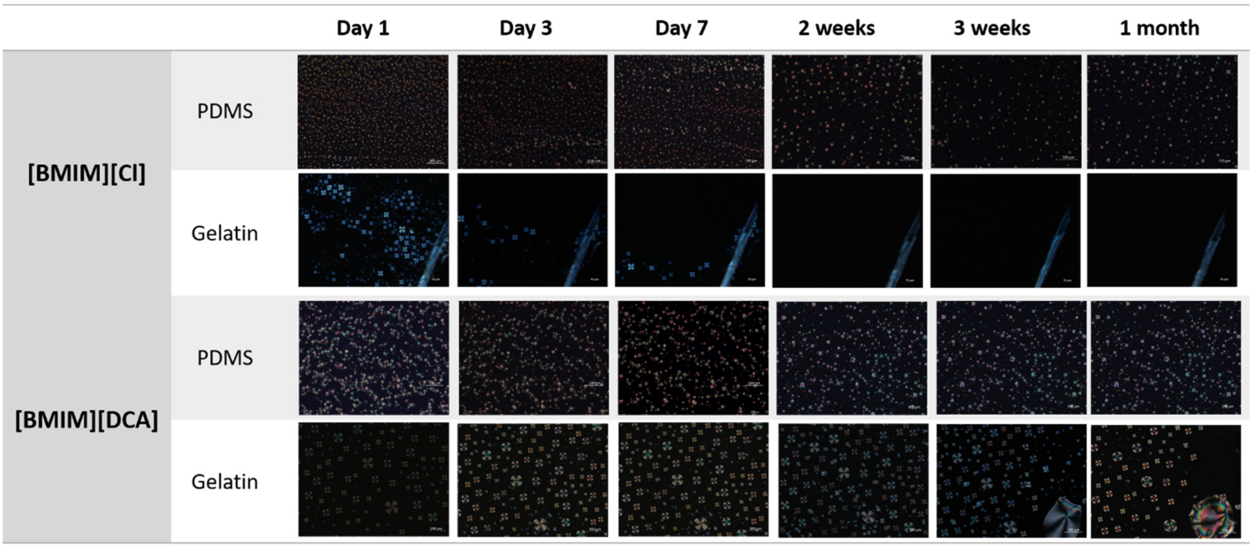

Figure S5. POM images of constant optically active areas obtained for the PDMS and gelatin sensing films produced from day 1 till 1 month after production. Images were obtained by Zeiss Polarized Optical Microscope (Zen 2.3 software), with 90° polarizers at 10x magnification.

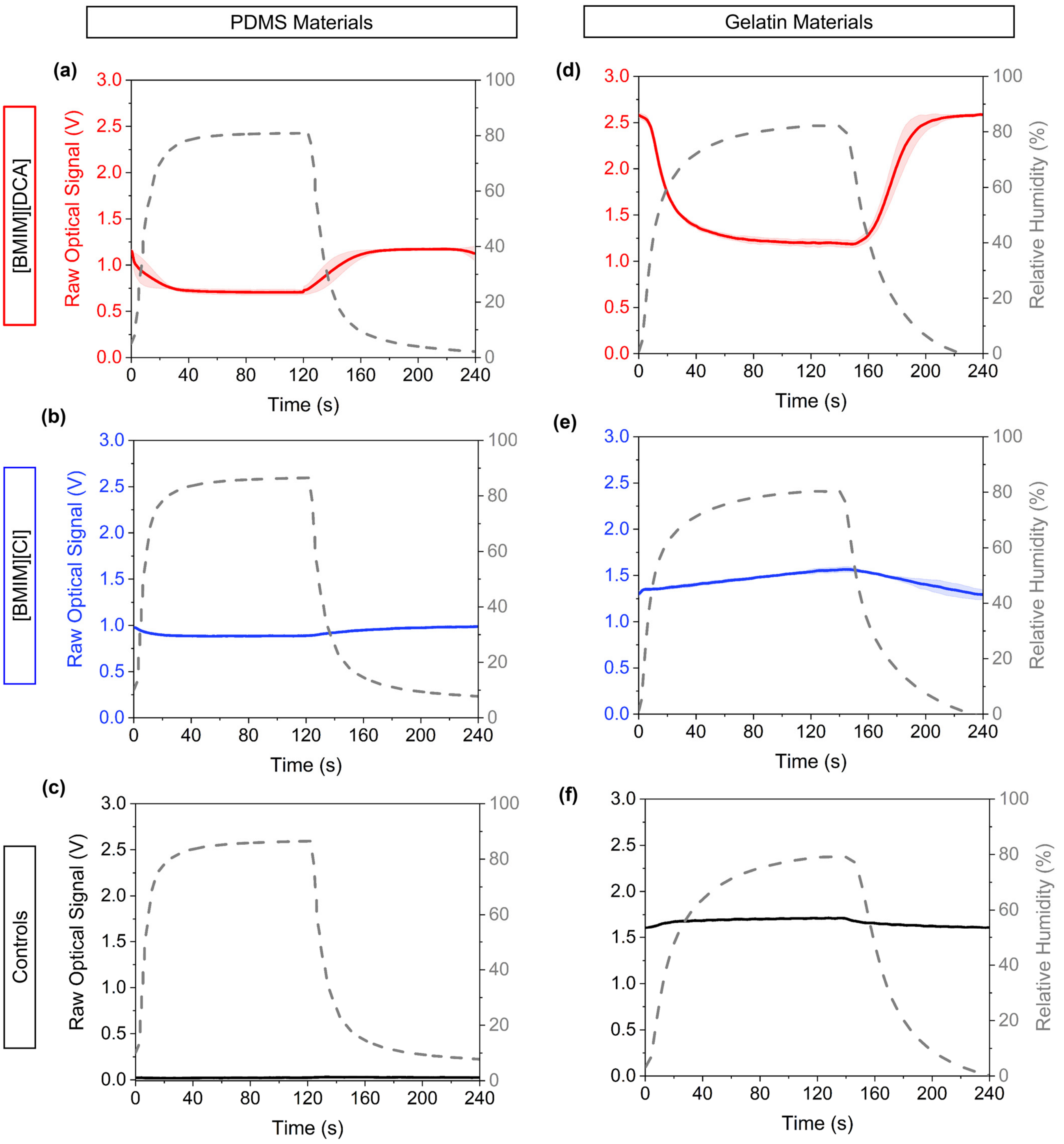

Figure S6. Raw optical signals of sensing films to a step change of relative humidity from 0% to 80%. (a**)** hybrid film composed by PDMS, [BMIM][DCA], 5CB; **(b)** hybrid film composed by PDMS, [BMIM][Cl], 5CB; **(c)** PDMS control film (without IL); **(d)** hybrid film composed by gelatin, [BMIM][DCA], 5CB; **(e)** hybrid films composed by gelatin, [BMIM][Cl], 5CB; **(f)** gelatin control (without IL) film.

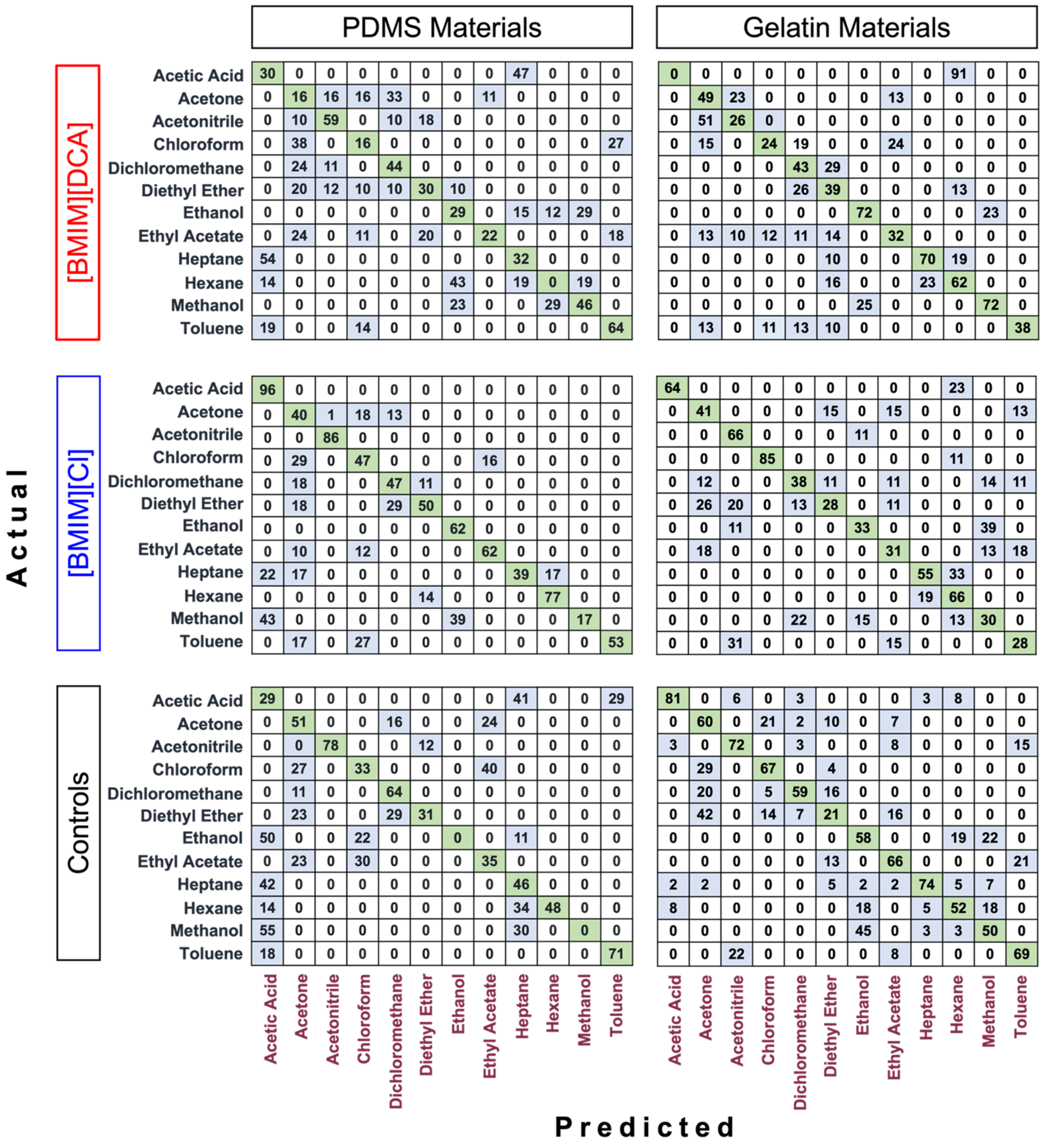

Figure S7. Optical e-nose confusion matrices obtained, illustrating the prediction results regarding 12 VOCs based on the signals obtained from the hybrid gel sensors with both PDMS and gelatin matrix hybrid sensors with two different ionic liquids ([BMIM][DCA] and [BMIM][Cl]). The green squares in the diagonal represent the percentage of correct predictions made by the classifier, and the lighter blue squares outside of the diagonal represent the percentage of incorrect prediction of a VOC.

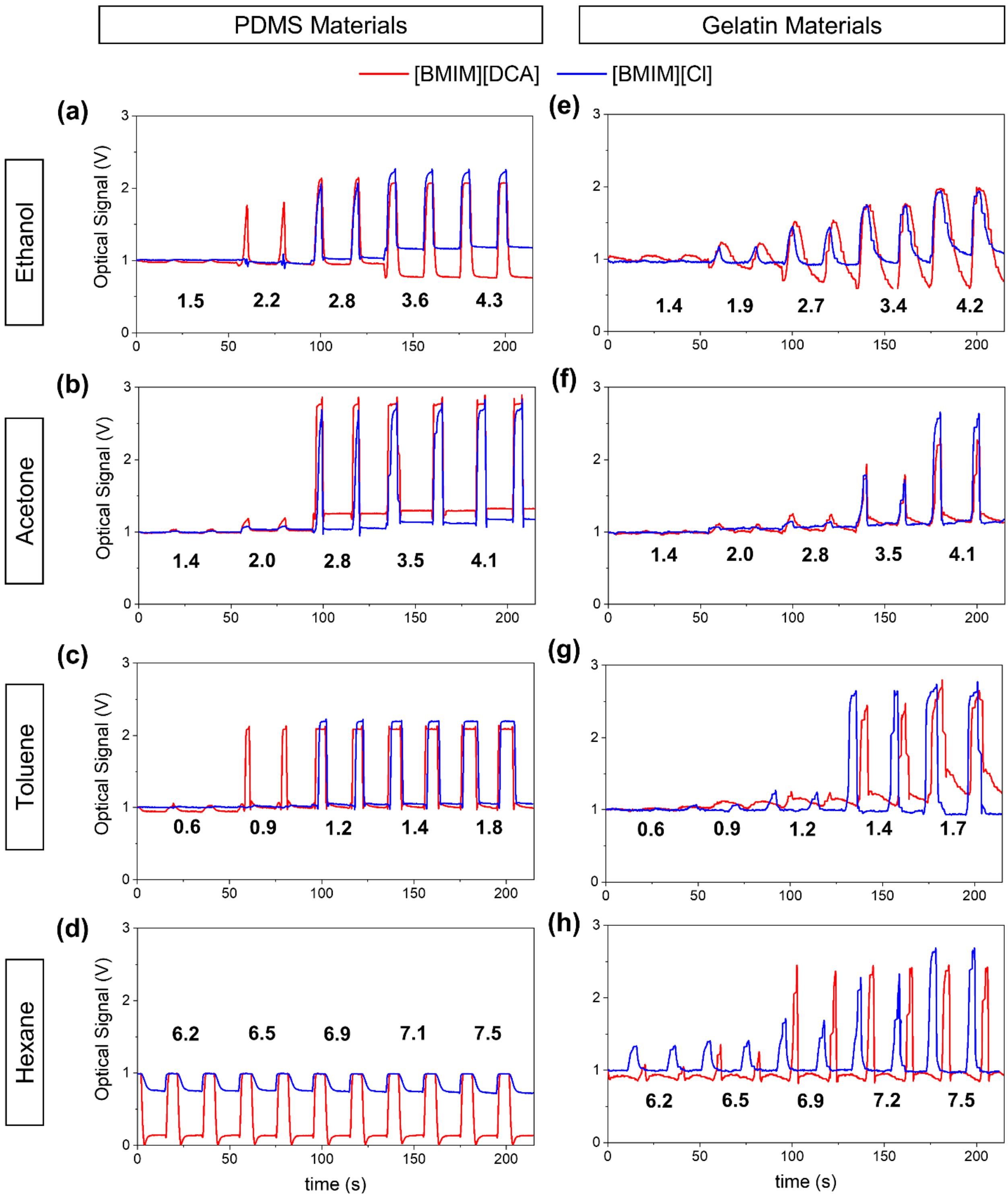

Figure S8. Variation of the amplitude of the optical response of hybrid films with the increase in concentration (indicated in %(v/v) for each plot), of ethanol, acetone, toluene, and hexane dissolved in humid (50% RH) nitrogen. **(a), (b), (c), (d)** Response amplitude of hybrid films composed by PDMS, the ionic liquid [BMIM][DCA] or [BMIM][Cl], 5CB and water. **(e), (f), (g), (h)** Response amplitude of hybrid films composed by gelatin, the ionic liquid [BMIM][DCA] or [BMIM][Cl], 5CB and water. (n = 2 exposure cycles to VOC for each concentration).

Table S3. Optical sensing performance of [BMIM][DCA] hybrid films of PDMS or gelatin at 50% RH.

Polymeric matrix	VOC	LOD (% v/v)	Triggering concentration (% v/v)	Saturation concentration (% v/v)
PDMS	Ethanol	1.03	1.65	3.24
	Acetone	1.16	1.99	2.57
	Toluene	0.44	0.70	1.20
	Hexane	n.d	1.76	3.60
Gelatin	Ethanol	1.00	-	3.13
	Acetone	1.70	4.03	5.02
	Toluene	1.00	1.06	1.60
	Hexane	n.d.	6.17	7.11

VOC, Volatile Organic Compound; LOD, Limit of Detection; n.d., Not detected; -, Not applicable.

Table S4. Optical sensing performance of [BMIM][Cl] hybrid films of PDMS or gelatin at 50% RH.

Polymeric matrix	VOC	LOD (% v/v)	Triggering concentration (% v/v)	Saturation concentration (% v/v)
PDMS	Ethanol	1.84	2.01	3.24
	Acetone	1.79	2.57	3.12
	Toluene	0.53	0.80	1.20
	Hexane	1.76	4.71	5.92
Gelatin	Ethanol	1.10	-	n.d.
	Acetone	2.20	4.63	5.74
	Toluene	0.53	1.12	1.77
	Hexane	2.27	6.17	8.23

VOC, Volatile Organic Compound; LOD, Limit of Detection; n.d., Not detected; -, Not applicable.
